# Physician privacy concerns when disclosing patient data for public health purposes during a pandemic influenza outbreak

**DOI:** 10.1186/1471-2458-11-454

**Published:** 2011-06-09

**Authors:** Khaled El Emam, Jay Mercer, Katherine Moreau, Inese Grava-Gubins, David Buckeridge, Elizabeth Jonker

**Affiliations:** 1 CHEO Research Institute, Smyth Road, Ottawa, K1H 8L1, Canada; 2 Paediatrics, University of Ottawa, Smyth Road, Ottawa, K1H 8L1, Canada; 3 Family Medicine, University of Ottawa, Bruyere Street, Ottawa, K1N 5C8, Canada; 4 College of Family Physicians of Canada, Skymark Avenue, Mississauga, L4W 5A4, Canada; 5 Epidemiology & Biostatistics, McGill University, Pine Avenue West, Montreal, H3A 1A2, Canada; 6 Direction de Sante publique du Montreal, Sherbrooke East, Montreal, H2L 1M3, Canada

## Abstract

**Background:**

Privacy concerns by providers have been a barrier to disclosing patient information for public health purposes. This is the case even for mandated notifiable disease reporting. In the context of a pandemic it has been argued that the public good should supersede an individual's right to privacy. The precise nature of these provider privacy concerns, and whether they are diluted in the context of a pandemic are not known. Our objective was to understand the privacy barriers which could potentially influence family physicians' reporting of patient-level surveillance data to public health agencies during the Fall 2009 pandemic H1N1 influenza outbreak.

**Methods:**

Thirty seven family doctors participated in a series of five focus groups between October 29-31 2009. They also completed a survey about the data they were willing to disclose to public health units. Descriptive statistics were used to summarize the amount of patient detail the participants were willing to disclose, factors that would facilitate data disclosure, and the consensus on those factors. The analysis of the qualitative data was based on grounded theory.

**Results:**

The family doctors were reluctant to disclose patient data to public health units. This was due to concerns about the extent to which public health agencies are dependable to protect health information (trusting beliefs), and the possibility of loss due to disclosing health information (risk beliefs). We identified six specific actions that public health units can take which would affect these beliefs, and potentially increase the willingness to disclose patient information for public health purposes.

**Conclusions:**

The uncertainty surrounding a pandemic of a new strain of influenza has not changed the privacy concerns of physicians about disclosing patient data. It is important to address these concerns to ensure reliable reporting during future outbreaks.

## Background

Providers under-report notifiable disease cases to public health agencies, sometimes by wide margins [1-26] (a summary of this literature is provided in additional file 1). Concerns about patient privacy are a key factor inhibiting disease reporting [6,11,13,19-21,27,28]. This is not surprising, given that high levels of concern about information privacy can negatively affect information disclosures [29-31].

Reluctance to disclose patient information for public health purposes exists [32], despite statutes in Canadian jurisdictions that mandate or permit reporting of personal health information (PHI) for public health purposes without patient consent [33]. Similarly, the US Health Insurance Portability and Accountability Act (HIPAA) Privacy Rule permits the disclosure of PHI to a public health authority without patient authorization [32,34-40].

It is not known whether such pre-existing privacy concerns would be diluted in the context of an actual pandemic influenza outbreak. Arguably, continued patient privacy barriers to reporting during a pandemic reflect a fundamental problem with data disclosure practices, which need to be addressed.

While there is always a balance between individual and collective rights, public health law provides sweeping powers in the context of a public health emergency. Some ethicists and policy makers have proposed that the rights of the individual, particularly the right to privacy of one's personal health information, should be subjugated to the collective interests of the community when this would help to prevent serious harm to that community [41-49]. For example, the Pandemic Influenza Working Group applies this principle as a value to guide ethical decision-making, noting that, "To protect the public from harm, health care organizations and public health authorities may be required to take actions that impinge on individual liberty" [42].

We conducted a mixed-methods study around the peak of the H1N1 outbreak between October 29-31 2009 to investigate what data Canadian family physicians are willing to disclose to public health agencies for influenza surveillance and reporting purposes, as well as the specific privacy issues that impact the disclosure of information. Based upon the results, we make policy and procedural recommendations to help eliminate barriers to data disclosure.

## Methods

### Intention to Disclose Personal Information

A number of theories and instruments have been used to measure and explain the relationship between information privacy concerns and individual behaviours [30,31,50,51]. Although none have been used in the context of disclosing patient information for public health purposes, we utilized them as a starting point to articulate a set of constructs, and a high level set of relationships among them. These guided our data collection and analysis.

A key one is Social Contract (SC) theory, which requires an equitable exchange and shared understanding about contractual terms and self-control over the course of a relationship [30,52,53]. In the context of information privacy, this theory suggests that an organization's collection of personal information is perceived to be fair only when the individual providing personal information is granted control over, and is informed about the organization's intended use of the personal information, and the disclosure of the information is perceived as equitable with some benefit back to the individual [30,52-57]. This leads to three important constructs which have been linked to the intention to disclose personal information: *collection*, *control *and *awareness*.

Collection, control and awareness affect the intention to release personal information through two intervening constructs: trusting and risk beliefs [30]. The reasoned action paradigm states that behaviour intention is a reliable predictor of actual behaviour [58,59].

The trust/risk/intention model states that in situations in which potential risks are present or perceived, trust plays an important role in predicting an individual's behaviour [60,61]. Trusting beliefs refer to the degree to which individuals believe that an organization is dependable to protect their personal information [62]. Risk beliefs refer to the high possibility for loss associated with the release of personal information to an organization [63].

Collection, control and awareness affect trusting beliefs [30]. Enhancing trusting beliefs will have a negative effect on risk beliefs (reduce risk perceptions), and will consequently facilitate intention to release personal information [30]. It has also been shown that trusting beliefs have a direct positive effect on the intention to release personal information [30]. Therefore, trust and risk beliefs are intervening factors that play an important role in facilitating or inhibiting the disclosure of personal information.

In our study we focused on exploring how the collection, control and awareness constructs affect intention to disclose information to public health for Influenza-Like-Illness (ILI)/H1N1 surveillance and reporting by affecting trusting and risk beliefs.

### Fields Collected for Influenza Surveillance and Reporting

Influenza is a disease under national surveillance [64] through many mechanisms, some of which vary from province-to-province.

The FluWatch program collects summary data from volunteer sentinel physicians across Canada [65]. The data reported weekly by participating physicians include: patient age in intervals, practice postal code, total patients seen, and the sentinel number, which identifies the reporting physician.

In addition, in some provinces, influenza is a reportable disease, with legislation requiring nominal reporting of patients with influenza, although nearly all reports are of confirmed cases from laboratories. Jurisdictions such as Manitoba [66], Nova Scotia [67], the Northwest Territories [68], and Ontario [69] collect: the patient's first name, surname, sex, full date of birth, full postal code, practice postal code, and the physician's name.

At the outset of the H1N1 pandemic, many provinces deemed cases of H1N1 influenza to be reportable, however, most reports continued to be of confirmed cases from laboratories, and follow-up was not possible for all cases as the number of reports increased. Subsequently, many jurisdictions required reporting for only hospitalized cases of H1N1 influenza. As examples, the case report forms contained the following fields across Alberta [70] and Newfoundland and Labrador [71]: patient's first name, surname, sex, date of birth, full postal code, pregnancy status, asthma history, COPD history, chronic heart disease history, and diabetes history.

Another H1N1-specific national surveillance program coordinated by the Public Health Agency of Canada [72] collects data directly from practice electronic medical records (EMRs) after their customization with new templates: age, gender, pregnancy status, presence of a chronic medical condition, such as cardiac, pulmonary, or diabetes.

In our study, we examined the willingness to disclose the specific fields noted above, since they were collected at some point during the 2009-2010 H1N1 pandemic.

### Study Design

This mixed methods study used a triangulation design [73,74] to explore what Canadian family physicians were willing to disclose to public health agencies, as well as the specific privacy issues that impacted the disclosure of information. With the assistance of a trained moderator, we conducted a series of five focus groups with family physicians in Canada, and asked each focus group participant to complete a questionnaire. The focus groups occurred during week 41 of 2009 (October 29-31), just as widespread influenza activity was starting to peak in Canada [75]. The study was approved by the Children's Hospital of Eastern Ontario Research Ethics Board prior to commencement.

In terms of deciding on an adequate number of participants and/or focus group sessions, there is no standard that is generally accepted. However, there are a few considerations that one should take into account when deciding on the number of participants required [76,77]. Firstly, there is the consideration that there are a limited number of viewpoints on a topic. Having a greater number of participants does not therefore necessarily entail a greater understanding of the topic [77]. The researcher can get a sense of how the group may be divided on an issue before commencing the focus groups by way of the published literature on the topic and/or by speaking with individuals in the group of interest [77]. Although opinions can vary, this would give the researcher an idea of when he/she has reached saturation; the point at which there is nothing new to be uncovered on the topic [76,78]. Krueger and Casey suggest starting with 3-4 focus groups to uncover the range of opinions on the topic [78]. If new ideas continue to appear, more focus groups should be undertaken, until the point of saturation is reached [78]. Once saturation has been reached, further interviews or focus groups will no longer be necessary nor particularly useful [76-78].

Secondly, the researcher's own ability to recall, process and understand the interviews needs to be considered [77]. As Gaskell points out, "the interviewer must be able to bring to mind the emotional tone of the respondent and to recall why they asked a particular question" [77]. Therefore, there is a limit to the number of encounters that the researcher will be able to recall in detail. For group interviews, Gaskell suggests that this limit would be 6-8 sessions [77].

A recent literature review has noted that the median number of focus groups conducted in health sciences research was 5, with a median of 32 participants [79].

Consistent with the recommendations in the literature and with precedents, we planned for five focus groups with a maximum of eight participants in each. We expected to reach a point of saturation in terms of new concepts identified within that. This expectation was partially driven by discussion with family physicians in preparation for the focus groups whereby we observed consistency in their views on data sharing for public health purposes.

### Study Sample

For the purposes of this study, purposive sampling was used [80]. Participants in the focus groups were recruited in advance of the Family Medicine Forum (FMF) in Calgary, Alberta via email invitation. The FMF is an annual conference of family physicians organized by the College of Family Physicians of Canada. The sampling strata used were years of practice (< = 5 years, between 6 and 10 years, and > 10 years), gender, location (rural vs. urban) and region of Canada (East, Central, West). The recruitment target was 8 participants for each of the five focus groups. Assuming a 33% no show rate, we aimed for 60 registrations. Invitations were sent to registered FMF delegates by email in advance of the conference, ensuring that all the strata were covered among those invited. Invited delegates self-selected to participate in the study.

The invitation email was sent by the College of Family Physicians of Canada. The email had basic information about the study and a link to a web page with an electronic consent form. The electronic consent form included a description of the study and its procedures, a description of withdrawal rights, data confidentiality, and information on how to contact the first author and the REB chair. Respondents could then enter their name and email address, and select the most suitable time for them for a focus group. By completing the form the respondents indicated their consent to participate.

A total of 37 Canadian family physicians participated in the study.

### Data Collection

The focus group sessions were conducted in a private meeting room at the FMF. At most two focus group sessions were held per day during the three day conference.

At the outset of each focus group, the participants were asked to complete a short questionnaire on paper to gather their views on data sharing. The questions included in both the questionnaire and focus group guide were constructed based upon the literature, our anecdotal experiences with the data sharing practices of physicians, and a pilot study with four physicians, who provided us with additional feedback and information.

The questionnaire and focus group guide consisted of two components. The first asked about willingness to disclose certain data elements to public health, and contained 15 items. These were the data elements that we found in current ILI/H1N1 case report forms used across the country. A set of 14 factors that we hypothesized would increase the willingness to disclose the above information were included in the second component of the data collection tools. We used a semantic differential scale [81] where a score of 1 meant "less willing to share data" and a score of 7 meant "more willing to share data" with public health.

For both the questionnaire and focus group guide, we formulated a scenario to ground the participants in a realistic example. In the scenario, the participants assumed that they had been requested to provide patient-level data to a municipal public health unit for the purpose of surveillance of ILI, specifically, H1N1 surveillance. As part of the scenario, we told participants that only individual level data on the patients who meet a particular case definition would be disclosed to the public health unit, as well as denominator totals. They were also informed that, in some jurisdictions, this reporting was mandatory.

### Quantitative Data Analysis

The questionnaire responses were transcribed into SAS. Ten percent of the questionnaires were randomly selected and cross-checked against the data entered in SAS by a different person than that who transcribed the data to check for systematic errors.

We first computed descriptive statistics on the questionnaire responses to understand the central tendency of the responses and variation to gauge consensus. The objective of that analysis was to document the fields that the participants were willing to disclose to public health, the factors that would change their willingness to disclose, and to understand if variation in participant responses can be explained by years of practice experience and gender.

Since some of the items in each section of the questionnaire are expected to be strongly related, we also grouped them into a smaller set to help with interpretation. To do so, we computed the mean absolute difference between each pair of items (105 pairs for the first part of the questionnaire, and 91 pairs for the second part). We used the difference instead of a product moment correlation for two reasons. First, the responses on two items could be strongly correlated but very different. It would not be appropriate to combine two items if they were different. Second, if the absolute difference were small, then it meant that the scores of the two items were close to the 45 degree positive diagonal, which implied a high positive correlation. The three criteria used for grouping elements were: (a) a mean absolute difference less than 1, (b) the grouping of items was meaningful, and (c) the group was consistent in its scoring (for example, if the difference between items between B and C was larger than the difference between items A and B, and A and C, then that three item grouping would not be consistent). Regarding criterion (b) the judgement was subjective, but it was almost always obvious. For example, grouping a high willingness to share the patient's first name with a high willingness to share a patient's COPD history would not be meaningful, but grouping the first name with the last name is more meaningful. Regarding criterion (c) we used the consistency ratio from the Analytic Hierarchy Process (AHP) to quantitatively evaluate the consistency among pairwise comparisons [82,83]. AHP is a widely used multi-attribute decision making methodology which utilizes comparisons by experts. Within AHP an analyst computes an index based on a matrix of pairwise comparisons and compares it to the value from a random matrix. The greater the consistency the closer the index is to zero. A ratio greater than 0.1 is considered representative of excessive inconsistency.

**Figure 1:**
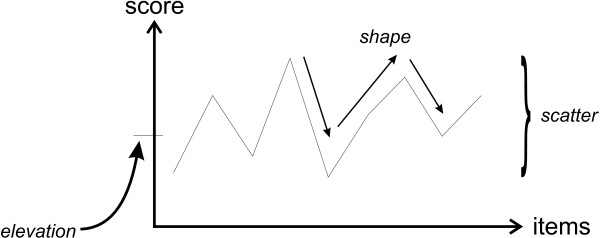
**Illustration of profile elevation, shape, and scatter**.

After grouping the items, we had a profile of responses for each participant on each of the two questionnaire sections. To understand whether the differences among participants were related to their years of practice and gender, we needed to define profile similarity measures. There are three ways to evaluate profile similarity: shape, elevation, and scatter, as illustrated in Figure 1[84]. Shape pertained to the pattern of responses to the items. Two profiles had the same shape if their responses were correlated. Elevation pertained to the proclivity of a participant to be willing to share information in the first part of the questionnaire, and their proclivity to change their willingness to share information in the second part. It was measured by using the mean score across all of the items. Scatter pertained to their variability in responses, and was measured as the range of item responses.

To assess differences in shape, we first grouped response profiles using a hierarchical clustering algorithm. The linkage method was Ward's [85]. The similarity measure among participants was the correlation coefficient. The emergent groups were compared on their mean years of experience, using a one-way ANOVA, and on gender using a chi-square test. This told us if the respondents differed by years of practice or gender in their response shape. The analysis was repeated using a Kruskal-Wallis one-way ANOVA, which provided a more robust test of medians across groups.

To assess if years of practice or gender were associated with elevation, we computed the correlation coefficient between the mean scores of each participant and years of practice, and compared males vs. females on mean scores using a t-test. Similarly, to assess if years of practice or gender were associated with scatter, we computed the correlation coefficient between the score range of each participant and years of practice, and compared males vs. females on score range using a t-test. All statistical significance tests were performed at a two-tailed alpha level of 0.05.

### Qualitative Data Analysis

The focus groups were audio-taped and transcribed verbatim. We used an approach informed by grounded theory [86,87] to analyze them. The objective of the analysis was to understand the reasons why the participating physicians were unwilling to share selected data elements. Data analysis began immediately after the first focus group session, and continued until a set of stable themes developed. Following each focus group session, the trained facilitator, assistant facilitator, and note taker engaged in a debriefing meeting, and conducted a cursory analysis of the audio recordings and field notes from the session. In this meeting, they revised, added, or removed selected open-ended questions in preparation for the next focus group session. As such, the questions used in each focus group were slightly different and became increasingly specific as the concepts needed to develop an understanding of physicians' perceptions about providing patient-level data to public health in the context of a pandemic disease outbreak became evident.

Using the constant comparison method [87], as well as the above-mentioned constructs from the literature, we also developed, modified, and agreed upon a coding scheme that embraced the themes presented in the data. Two research team members trained in qualitative research methods independently coded the transcripts using NVivo software and an inductive process. Once the members completed their independent coding, they shared and compared their coding, as well as discussed the accuracy of it. At this point, they modified, merged, or eliminated codes and revised their analyses.

## Results

### Participants

Forty three physicians registered to participate in the focus groups, and 37 showed up. This amounts to an 86% participation rate, which is slightly higher than the participation rate we had assumed in the design (40/60 at 66%).

The 37 participants had a mean of 16.3 years of practice experience, with a maximum of 43. The overall family physician population summary data from the 2007 National Physician Survey (NPS) [88] reported that the mean years of practice experience was 19.6, with a maximum of 55 years.

There were 24 female (65%) and 13 male (35%) participants. The 2007 NPS reported 60% of family physicians are male and 40% female.

The distribution by province was: 18 from Western Canada (49%), 14 from Central Canada (Ontario and Quebec - 39%), and 5 from Eastern Canada (13.5%). The 2007 NPS reported that approximately 35% of family physicians practice in Western Canada, 59% in Central Canada, and 9.3% in Eastern Canada.

Compared to the physician population, our participants had slightly less practice experience, and were more likely to be female. Furthermore, physicians from Central Canada were under-represented whereas physicians from other regions of the country were over-represented.

### Quantitative Results

The results for the questions on which types of fields the physicians would be willing to disclose are provided in Table 1. There was strong reluctance to provide patient names, a weak willingness to provide dates of birth, postal codes, and initials, a moderate willingness to reveal their own names, the number of patients in their practice, and clinical information about the patients, and a stronger willingness to share patient sex, the total number of patients seen during the reporting period, and the practice postal code. The least consensus was in their willingness to share the patients' postal codes, date of birth, initials, and the most consensus was on their willingness to disclose the patients' sex.

**Table 1:** Descriptive summary of the responses on the type of information that the physicians would be willing to share.

	Information to Share	Mean	Std. Dev.
**A1**	Patient's first name	2.8	2

**A2**	Patient's surname	2.5	2

**A3**	Patient's initials	4.3	2.3

**A4**	Patient's sex	6.6	0.6

**A5**	Patient's full date of birth	4.2	2.3

**A6**	Patient's full postal code	4.8	2.5

**A7**	Indication of whether the patient is pregnant	6.2	1.3

**A8**	Patient's diabetes history	5.7	1.7

**A9**	Patient's asthma history	5.7	1.7

**A10**	Patient's COPD history	5.7	1.7

**A11**	Patient's CHF history	5.7	1.7

**A12**	Your practice postal code	6.2	1.4

**A13**	Your name	5.22	2.1

**A14**	Total number of active patients in the practice	5.7	1.7

**A15**	Total number of patients seen during the reporting period	6.2	1.3

A score of 1 means "not willing to share", a score of 7 means "very willing to share", and neutral value was coded as 4

If we assume that the willingness to disclose is positively associated with perceived sensitivity of the information, then there was the most consensus on the least sensitive fields, and less consensus on the most sensitive fields. This would be the expected pattern.

We grouped the following items: (a) items A1 and A2, (b) items A7 to A11, and (c) items A14 and A15. Those in group (a) pertain to the patient's name, and the participants didn't see much difference between disclosing the first and second name. The items in group (b) pertain to underlying conditions. The participants treated all of the underlying conditions equally in terms of willingness to disclose and did not provide an indication that they differ in their sensitivity. The items in group (c) pertain to summary information about the practice. Group (b) had a consistency ratio below 0.1 (consistency does not apply to the other groups with only two items).

Table 2 shows the willingness to share less/more information under certain conditions. The factors that had little impact on their willingness to disclose information to public health were: linking the data to other data sets, whether the public health unit was provincial, federal or municipal, and the type of condition or disease that was under surveillance. The factors that had the largest influence on their willingness to share information were: their commitment not to share the data with other parties, and whether the reporting was mandatory. Other factors that would have an important effect on their willingness to disclose information included if the data was de-identified, if their Colleges endorsed the disclosure, if feedback was provided, if the disclosure was only for a limited period and afterwards the data would be destroyed, and if a research ethics board approved the data collection. None of the factors would actively dissuade them from sharing data, but that was largely a function of the wording of the questions.

**Table 2:** Descriptive summary of the responses on the questions on the factors that would influence the physicians' data sharing practices.

	Factors Influencing Sharing	Mean	Std. Dev.
**B1**	The data collector was a provincial ministry of health or agency rather than a municipal public health unit	4.4	1.3

**B2**	The data collector is Health Canada/Public Health Agency of Canada rather than a municipal public health unit	4.6	1.4

**B3**	The data will be linked with other administrative and clinical databases by the public health unit	4.2	1.4

**B4**	The disclosure of the data to the public health unit has been approved by a research ethics board	5.8	1.3

**B5**	The data will be appropriately de-identified before it is sent to the public health unit	5.8	1.6

**B6**	A commitment is made by the data collector not to share the data with any other third party	6	1.4

**B7**	The disclosure of the data to the public health unit is mandated by law	6.4	1.2

**B8**	The disclosure of the data is for a limited duration and will cease afterwards	5.7	1.3

**B9**	The public health unit informs the public (e.g., through their web site or newspaper advertisements) that this type of information will be collected from their family physician	5.1	1.5

**B10**	The disclosure of the information has been cleared/approved by the provincial college of physicians and surgeons	5.8	1.4

**B11**	The disclosure of the information is endorsed/supported by the CFPC and/or the provincial college of family physicians	5.8	1.4

**B12**	The public health unit will provide regular custom feedback reports to your practice about disease activity in your area and patient risk	5.7	1.2

**B13**	The data will be used for research purposes by the public health unit collecting the data (rather than just for public health surveillance)	5	1.4

**B14**	The disease being monitored is different (i.e., it is not influenza but say an STD or a chronic disease)	4.3	1.3

A score of 1 means "less willing to share data", a score of 7 means "more willing to share data" with public health, and a score of 4 means that the factor has no influence on their sharing decisions

The following items had a mean absolute difference smaller than one point and were grouped: (a) B1 and B2, (b) B5 and B6, and (c) B10 and B11. The items in group (a) pertain to the jurisdiction of the public health agency. The physicians did not differentiate between federal or provincial jurisdictions. The items in group (b) pertain to exerting control over the data through de-identification, and limits on sharing with third parties, which they perceived as having equal impact on willingness to share information. The items in group (c) pertain to independent oversight, irrespective of who provides it.

We did not find any statistically significant differences nor associations among the participant profile shape, elevation, or scatter and the physicians' years of practice experience and gender. This means that variation in years of practice experience and gender do not explain variations in the participant responses to the questions.

### Qualitative Results

The qualitative findings are organized into six themes. Each theme presents a coherent issue, although it may cover multiple constructs.

### Physician-Patient Relationship

Some participants believed that the government and their requests for information and data were interfering with patient-physician relationships. Many agreed with one physician, who stated: "*We have to remember that as family doctors, we have a real responsibility to the patient...What are the patients going to start thinking about, you know, where are they getting this information? Who is telling the government or the newspaper about me? Or my family or the neighbour? So I think we have to really, you know, be careful of the patient [who] comes to us, they trust us. They want to have trust in us, and there is nobody else that can fill that role other than the family doctor."*

In particular, this issue of trust and the patient-physician relationship was of concern to physicians serving aboriginal or minority populations: "*I mean, you may find, depending on ethnicity of the people we work with, whether they are First Nations or whether they are, I work with a fairly ... large immigrant population, and there's sometimes complete lack of trust, you know, depending on, especially if you start tracking demographic data. People sort of start to wonder, right, what's happening."*

Our participants were also concerned about patients possibly complaining about them or suing them, if they inappropriately disclosed information. One participant told a story of being contacted by a patient's lawyer, after data was abstracted from the patient's chart and entered into a provincial registry.

### Physician Confidentiality

In the focus group discussion, a not insignificant amount of mistrust between physicians and "government authorities" was also apparent. Specifically, some participating physicians were concerned that the data that they provided to public health was being, or would be linked to their performances as physicians: "*I could see some [physicians] being a bit concerned...if [data] were linked to some performance evaluation, like, are you not picking up enough..." *and "*I have a few letters from the ministry over the years, telling me that I was more than two standard deviations outside the norm, but I do palliative care, so I do too many house calls apparently, because I do home palliative care, and you get a couple of those letters, and they ask you to justify your practice, and it makes you feel a little bit paranoid about, you know, big brother watching."*

Furthermore, the weak data handling practices of organizations that collect data from physicians have amplified this lack of trust: "*I think there is a great deal of mistrust between physicians and the bodies that collect data. In [our province], every time we write a prescription, and the patient takes that prescription to a pharmacy and the prescription is filled, it enters to the [central pharmacy] database. And the provincial government has been working very hard to link the medical services plan, physician billing data, [central pharmacy database], and hospital data. And they ran into some really serious privacy concerns [linking] those administrative databases at the patient level, or even at the physician level without consent... The problem is there has been several scandals in my province where hard drives, or laptops or documents that should have been shredded have been discovered, turned over to the media or you know have been, have been not adequately looked after ... you have one or two of those mistakes and immediately everybody says, oh well, you know, we can't trust that all this data that we're sending up the food chain all the time is actually being properly managed."*

Poor data handling practices and breaches by government data recipients, even if they are not at public health agencies, have made some participants concerned about disclosing health information to public health.

### De-identification and Notice

When disclosing data to public health where direct contact with the patient would not be necessary, for example, indicator-based surveillance efforts for situational awareness, the general consensus was that the data needs to be de-identified. Many of the physicians reiterated the fact that they "*would be uncomfortable providing patient identifying data ... if it could be linked personally to any of [their] patients, that is, [they] would be uncomfortable, unless it was ... totally anonymous."*

Throughout the discussions, there was also variation in how patient information should be de-identified (for example, including or not including postal codes) and as such, the need for clear guidelines on what constitutes de-identified patient information was expressed.

De-identification by itself, however, was not seen as enough. Engaging the patients in the disclosure decision was suggested as *"the patient needs to have a role in [the disclosure]. They need to say, that is okay"*. Several of the participating physicians suggested the need for obtaining individual consent by stating that *"confidentiality and privacy is not a mass issue, it's an individual issue, so the individual should be informed specifically" *whereas others stated that, *"There has to be explicit consent, it cannot be implied consent. Just because I saw this doctor, the information will be transferred to public health. That's not acceptable"*. That said, many physicians also stated that obtaining individual consent was generally seen as not practical as "*most of [them] don't have the time to spend explaining patient consent for something like this*." However, other participants thought that the consent processes could be more general, for example, posters in the physicians' offices notifying patients that their de-identified information may be released to public health agencies. This was seen as a more practical alternative to obtaining individual consent.

Where reporting of influenza or ILI is mandated, public health agencies require the disclosure of detailed information about the patient, such as their name and address. Some of our participants were uncomfortable providing such detailed information: *"I think the bottom line for most family physicians is we will not share names, addresses, or phone numbers, period, without individual patient consent,... for the most part, what we would probably be willing to share is the de-identified, already anonymized aggregate data. I don't think it's realistic to expect any family physician to breach confidentiality and share information." *This is consistent with the literature showing under-reporting for notifiable diseases. Of course, contact tracing or other investigations that require public health to communicate directly with the patients would not be possible without identifying information. In such cases, some of the factors discussed below (such as articulating the purpose, data sharing agreements, and patient benefit) would be important to address the physician concerns about disclosing patient-identifying information.

### Constraints on Disclosure

Physicians were reluctant to provide certain types of patient data because they did not know for what purpose the data were being, or would be, used for and who it might be shared with afterwards. For example, one participant said, *"Well I'm concerned about how they might use it....I mean if they use the data for their own internal operations, that's fine, but if they want to share it with a non-healthcare agency, I don't think that's fine"*. Likewise, another stated, *"We should have the right to know why they are using [it], and what are the reason[s] for collecting and what the data is for."*

The majority of participating physicians also stated that if they knew how the data that they provided was being used, they would be more motivated to provide additional data: *"If you see the end of it. The benefit from the data, so you see the results of the data being processed, then you might be more willing to join in to helping the gathering of the data"*.

Participating physicians stated that they would like to have a comprehensive data sharing agreement with public health agencies prior to disclosing any patient-level data. Included in this data sharing agreement, physicians would like to see information about how the data would be used (for what purpose), how long the data will be kept, as well as who will have access to it. These agreements would not only provide the physicians with, *"assurances that data will not be used for things beyond the use of the agency it's going to" *but also help to *"get physicians involved"*, and make them feel more *"comfortable to comply"*.

### Patient Benefit

Participating physicians questioned how the data requested by public health agencies would benefit *their *patients: *"What benefit would it offer to the individual [patient] for us to release their private health data? What does public health have to offer them that's going to be of benefit to them?" *and *"Does public health really need to know this information? And can they show us a very clear pathway of how the information can either improve patient care or patient outcomes?" *A stronger incentive than general benefit to the population was also seen as important. For example, one participant stated "*Patients look to us to be their expert and to be their advocate. So, we, as physicians, are happy to provide the information to public health for the greater good for public health, for the country as a whole, but on the other hand, in return we need to be given something of value that we can give to our patients, so we don't feel like we are compromising our patients." *Another stated, "*As long [as the data] is going to benefit the patient, it shouldn't just be for the sake of research or somebody's PhD thesis. It has to benefit the patient."*

In the specific context of the H1N1 pandemic, participants questioned the benefit to their patients of disclosing information: "*In a situation where, medication is unlikely to be a great deal of benefit, the immunization is arriving, you know, a bit late for it to be useful, what benefit would it offer to the individual for us to release their private data to public health? What does public health have to offer them, that's going to be of benefit to them? A whole bunch of nothing, right ? So, so we are not in a situation where we can therefore justify releasing that information."*

This suggests that being clear about the uses of the data is a necessary, but insufficient condition for the physicians to be willing to disclose patient information. The link to patient benefit needs to be articulated clearly when information is requested, and has to be adequately demonstrated.

### Communication and Feedback

Historically, physicians have indicated that they have not received sufficient feedback from public health agencies [7,19,89], and many participants reiterated this fact in the focus group discussions. This insufficient communication often results in physicians who are unmotivated to provide the data. The lack of feedback to physicians was articulated by statements, such as: "*The region I'm in, we don't, we have to call public [health], we have to harass them to get any kind of information. If I happen to not watch the news that day, I'm less informed than my patients, as to what's happening, you know, we feel like we have to run after them in order to get any information, and that's specific to this, you know, this recent issue. But generally, I don't feel like we get regular updates as to what is happening in the community. It's like it is always a matter of take, take, take from us. And rarely do we get to see what comes of that"*. Moreover, for those who have provided data to public health: "*It's just a matter of, or the fact that we keep giving them stuff, and we don't get anything in return, we get no communication in return."*

Therefore, even if the purpose of the collection is clear, and the link to patient benefit is clear, the feedback to physicians has to actually regularly happen afterwards. In the context of the H1N1 pandemic, a typical comment was, "*I think the only thing I have seen with the H1N1 thing is that we've had fairly poor communication from public health to physicians, I don't know if anyone else has had that, but even our, our, our, our provincial head of the public health wrote us all a letter, and said if you got any concerns, you can email me or fax me, but then he didn't provide any phone numbers or fax"*.

The format of the feedback is also important. For example, in terms of information formatting, physicians would like, *"Large format. Simple, easy to understand numbers"*, *"concise, something... [they] can read"*, and *"One page. [We'll] read one page"*. Several physicians also stated that they would like to control the quantity, type, and frequency of feedback and information they receive, and as such, an Internet-based information repository that is regularly updated with local information would be highly beneficial. For the most part, the participating physicians are particularly interested in obtaining clinically relevant information that they can use to provide better service to their patients: "*So, clinically relevant information, so, for example, if [public health agencies] are seeing a larger volume in your community, that's helpful to me, because maybe, for example, mine, it might look different. And public health is in the unique position that they are receiving all this information, but they may be disseminating numbers, and what we want to know is clinically relevant information. If you have that information, is it possible for you to package it in a way that for clinicians it is useful to us?"*

On the other hand, there was also concern expressed about too much repetitive information being provided by public health [90]. For example, one participant noted "*I'm on the other side where I'm being bombarded with useless flyers, useless [expletive] flyers, like I don't want to open, like I see the email address and I'm at the point where I kind of briefly open it, then delete it ... it's useless and it takes so much time. They add in all of their letterhead, all of the little pictures that take, depending on where you are, you are using dial up internet that takes forever to download, ... this is not useful. This is what we mean by useful information. Just text, that we can get anywhere, factual information and updates as to what's happening, [the] repetitive hand washing posters are not helpful. I got it, I got it."*

## Discussion

### Summary and Recommendations

**Figure 2:**
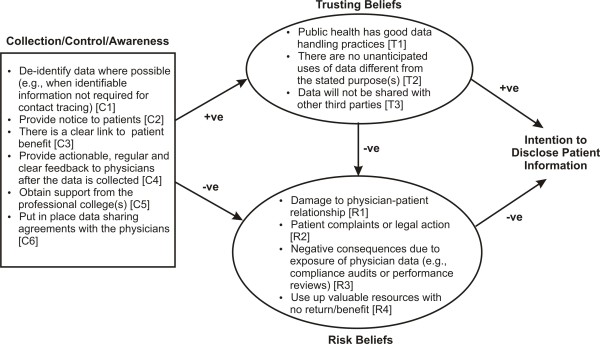
**A conceptual model summarizing our results**. The "+ve" and "-ve" indicate the direction of the relationship. The codes in the square parentheses are used to reference elements of the model from the main text.

Despite calls for weighting the public good more heavily in the context of a pandemic [41-48], physicians did not see it that way: their privacy reservations remained quite strong. Our results can be represented in the conceptual model of Figure 2. The collection/control/awareness box includes a list of recommended actions, mostly for public health agencies who wish to collect the data from family physicians. These actions would have a positive impact on the trusting beliefs that we found in our study, and a negative impact on the risk beliefs that we found. Trusting beliefs positively influence the intention to disclose patient information, and risk beliefs negatively influence it. Higher trusting beliefs reduce risk beliefs.

### Trusting Beliefs

Three factors increased the degree to which the physicians perceived public health as dependable to protect health information, and that make up the trusting beliefs construct. The first is the degree to which public health has good data management practices in place (factor T1). Stories in the press about data breaches at, or by public health agencies and health departments [91-97] reduce trusting beliefs. Second, the data must not be used for any purposes other than those for which it was originally collected from the physicians (factor T2). Even though privacy legislation in some jurisdictions does permit uses different from those under which the data was collected [33], the exercise of such discretions may erode trusting beliefs. Third, the data disclosed by the physicians is not shared with other third parties (factor T3).

### Risk beliefs

Four factors increase the possibility of perceived loss from disclosing the information, and these make up the risk beliefs construct. The first is damage to the physician patient relationship (factor R1), which can result in patients changing their behaviors to protect privacy. There is evidence that patients will adopt privacy protective behaviors when seeking care, if they have concerns about how their personal health information will be used or shared. For example, between 15% and 17% of US adults have changed their behavior to protect the privacy of their PHI, doing things such as: going to another doctor, paying out-of-pocket, even when insured, to avoid disclosure, not seeking care to avoid disclosure to an employer, giving inaccurate or incomplete information on medical history, self-treating or self-medicating, rather than seeing a provider, or asking a doctor not to write down the health problem or record a less serious or embarrassing condition [98-101]. Privacy concerns have caused individuals to not be totally honest with their health care provider [102]. More than a quarter of teenagers indicated that they would not seek out health care if they had concerns about the confidentiality of their information [103]. A survey of service members who had been on active duty found that respondents were concerned that if they received treatment for their mental health problems, it would not be kept confidential and would have a negative impact on future job assignments and career advancement [104]. It has been estimated that 586,000 Americans did not seek earlier cancer treatment, and 2.07 million Americans did not seek treatment for mental illness due to privacy concerns, and fear of harm to job prospects or other life opportunities, if the information was not kept confidential [105]. In a survey of physicians in the US, nearly 87% reported that a patient had asked that information be kept out of their record, and nearly 78% of physicians said that they had withheld information from a patient's record due to privacy concerns [106]. Public opinion surveys in Canada found that, over the prior year, between 3-5% of Canadians have withheld information from their provider because of privacy concerns, and 1-3% have decided not to seek care for the same reasons [107]. Furthermore, between 11% and 13% of Canadians have, at some point, withheld information from a health care provider because of concerns over whom the information might be shared, or how it might be used [108-110], with the highest regional percentage being in Alberta, at 20% [108]. Similar results have been reported by the Canadian Medical Association [111]. An estimated 735,000 Canadians decided not to see a health care provider because of concerns about the privacy of their information [112]. Specific vulnerable populations have reported similar privacy protective behaviors, such as adolescents, people with HIV, or who are at high risk for HIV, women undergoing genetic testing, mental health patients, and battered women [113].

The second risk beliefs factor (R2) is that of patient complaints or legal action if they are surprised by, or disapprove of how, and to whom their information is being disclosed. Disclosures by providers without individual patient consent have resulted in tortious or contractual claims of invasion of privacy, breach of confidentiality, or implied statutory violations under state law [114]. Concerns about their own privacy, and how their patient information could potentially be used against them is the third risk beliefs factor (R3) [115]. The lack of time to complete case report forms for public health has been an on-going problem [6,11,13,17,116]. The risks of investing scarce time, and disclosing their patients' information without getting back regular, actionable, and clear clinically relevant information from public health makes up the fourth risk factor (R4). This risk factor reflects the balance between costs and benefits: physicians would perceive less risk if the costs to provide information were mitigated by benefits. Of course, the lower the costs the better, or if someone else can cover these costs.

### Recommended Actions

Based upon our results, we can make six recommendations of actions that public health agencies can take to influence the trusting and risk beliefs of family physicians. These would then, in turn, have a positive impact upon their willingness to disclose patient information. The recommendations make up the collection/control/awareness constructs on our conceptual model.

Canadian public opinion research has found that patients prefer to be provided for some form of opt-in or opt-out consent before their personal health information is used for secondary purposes (such as public health) [117-123]. While some of our study participants advocated obtaining patient consent for disclosing their information to public health, it was generally seen as not practical [124], especially if there is a pandemic with many cases requiring reporting. Automation may ease the reporting burden [125-128]. For example, electronic transmission of disease information from family physician practices participating in sentinel networks to public health units for disease surveillance is becoming common [129-131], and the deployment of EMRs is also growing [132-140]. Furthermore, the ability to electronically submit syndromic surveillance data to public health is one of the EMR "meaningful use" criteria in the US [141]. It remains an empirical question whether, in practice, time not spent on actual reporting due to automation could be used instead to obtain consent. Even if consent can be obtained with minimal impact on time, there is compelling evidence that consent results in biased data, because consenters and non-consenters differ on important demographic and socioeconomic characteristics [142].

In reality, patients are only given notice about data being reported to public health rather than being offered an opt-in or opt-out consent. For instance, when disease notification is mandated, physicians are reporting fully identifiable information, and it is expected that public health will follow-up with the patients. According to the participants in our focus groups, it is common practice among reporting physicians to inform the affected patients that public health has been notified, and that they may initiate contact tracing and disease control efforts with them directly.

In situations where collecting identifiable patient information (such as patient names) is not mandated, public health may still want those fields to link with other data sets [49]. Informing the patients during the encounter would likely require explanations of why this is happening, and who the data is going to. This would start to resemble obtaining consent in terms of effort required, and raises the question of what to do if the patient objects. Based upon the views of our participants, not informing the patients at all would likely not be acceptable. Under such circumstances, it may be possible for public health to deploy secure linking methods that do not require the disclosure of identifiable information [143].

Not all surveillance efforts need personally identifiable fields, (i.e. patient names and addresses), for example, as in indicator-based surveillance programs. In such cases, de-identified patient data can be disclosed. The public is more comfortable with their health information being used for secondary purposes if it is de-identified at the earliest opportunity [118,119,122,144-148]. There is no legislative requirement to obtain consent if the information disclosed is de-identified. Recent indicator-based surveillance efforts have required the de-identification of patient data before it was disclosed [72,149,150].

Therefore, it is recommended that, wherever possible, data disclosed by physicians should be de-identified (factor C1) and that public health should provide notices for physicians to post in their offices informing their patients that de-identified information will be disclosed for specific public health purposes (factor C2). Furthermore, to the extent that public health agencies can use the media to educate the public that their health information is being disclosed for public health purposes, then this could be another method of providing notice.

De-identification must mask the origin of the data as well. This will hide the practice which generated the data, and would address the physicians' concerns about how they themselves will be affected by data sharing. There has been concern expressed about the impact upon the physicians themselves of disclosing patient information [11,151], in the context of being targeted by marketers, for example, or if the physician's information is being used to evaluate compliance with clinical practice guidelines, compliance with pay for performance programs, and concerns that their income could be affected if complete patient data from their practices was disclosed [152].

The location of the reporting practice is important to identify geographic patterns in a disease outbreak. To ensure that location is still known, even if the practice identity is masked, practices can report as a group, with each group consisting of a set of geographically adjacent practices. A protocol and system for the secure computation of case counts from physician practices for the purpose of surveillance has been developed [115]. Reporting as a group of practices can potentially make it more difficult for public health to identify the source of unusual spikes. This protocol therefore allows the public health unit to rank the data sources by their counts and hence initiate additional focused data gathering.

Our participants also indicated that they would be more motivated to disclose data if they were provided with actionable, regular, and clinically relevant information back from public health agencies that could be beneficial for their patients (e.g., timely alerts when there are local outbreaks, instead of first getting the information from the media and their patients). The lack of information back from public health agencies has been an on-going issue inhibiting willingness to disclose data [7,19,23,125,153-155]. This is captured by the benefit factor C3 and feedback factor C4. Such feedback would provide a tangible short-term benefit to the physicians in exchange for the data. There do exist examples of context specific public health alerts integrated with EMRs [156].

The support of the professional Colleges could be helpful in two respects (factor C5). First, they could perform the legal review of a standard data sharing agreement with public health to ensure that it adequately covers all the important elements noted above, and protects the interests of the physicians. They can also provide external validation that the data to be collected is indeed required for the stated public health purposes, and is the minimum necessary to achieve these purposes. The "general limiting principles" in privacy laws stipulate that personal information should be collected, used, or disclosed only where no other information will serve the purpose [157].

Although some participants indicated that they would like to see research ethics review boards endorsing disclosures to public health, this is not likely to be practical, given that many REBs are already under-resourced. Furthermore, the underlying concern is with independent external validation, which can be provided by the professional Colleges.

Data sharing agreements between physicians and public health should be put in place (factor C6). Agreements would be beneficial for individual case reporting, as well as surveillance for situational awareness. The agreements would explain why the information is being collected (for what purpose), how it will be used, who would have access to that information, limits on disclosures to third parties, restrictions on attempts at re-identification for de-identified data, how long it will be retained, and provide assurances that good information security practices will be put in place to manage the data. Although putting such agreements into place with a large number of physicians and physician practices would be time consuming, this would be a good investment to increase willingness to share information and increase reporting.

## Limitations

Our focus group study collected data from only 37 family physicians. While this can be considered a small sample, we did reach a point of saturation during the study whereby later focus groups were not contributing new factors to the evolving model of information disclosure. Therefore, it is not obvious that the addition of more focus group sessions would have provided additional information. Furthermore, the size of our focus groups and their number is consistent with the considerable precedent in the health sciences research literature [79] and recommendations in the qualitative research literature [76-78].

Compared to the physician population, our participants were different in terms of years of practice experience, gender, and practice location. However, we did not find years of practice experience or gender to be associated with the questionnaire responses.

During three of the focus groups the Canadian SARS experience was mentioned. SARS affected physicians in Central Canada mostly. Therefore, despite the under-representation from Central Canada, public health concerns that were most pertinent to that geographic area were still well represented during the discussions.

An important limitation of our study was that it was conducted with family physicians in Canada. Our resultant model will not necessarily apply without modification to other jurisdictions with different socio-cultural and legal contexts, and to different specialties. However, we have provided a testable theoretical model as a result of this work, and this model provides a basis for future research to examine the factors that affect the disclosure of information for public health purposes. Whether this theoretical model is generalizable beyond the specific context that we studied remains an empirical question.

The pandemic H1N1 influenza outbreak had relatively mild morbidity and mortality, which may explain why the participating physicians retained strong concerns about data disclosures of patient data. It is arguable that had the outbreak been more severe, or had more serious health consequences, their views on privacy may have shifted more towards greater willingness to share data with public health.

Implementing our recommendations would facilitate disclosure of patient information for public health purposes, but there are other barriers beyond privacy that would need to be addressed, for example, resources for reporting. Therefore, dealing with the privacy concerns may not be sufficient by itself to improve reporting to public health.

## Conclusions

Privacy concerns by providers have been a barrier to disclosing patient information for public health purposes. This is the case even for mandated notifiable disease reporting. We conducted a mixed-methods study with Canadian family doctors to understand the privacy barriers which could potentially influence family physicians' reporting of patient-level surveillance data to public health agencies during the Fall 2009 pandemic H1N1 influenza outbreak.

We found that Canadian family doctors do have concerns about patient privacy and about the disclosure of information that may be reflective of their own performance. Privacy is only one of a number of factors that affects their willingness to disclose patient data. We have formulated a conceptual model explaining how certain actions can facilitate the disclosure of health information by family physicians. The model contains a number of testable hypotheses, and also provides concrete recommendations for activities that are expected to increase the physicians' intention to disclose patient information to public health. In future work, we plan to perform confirmatory studies of this conceptual model.

## Abbreviations

AHP: Analytic hierarchy process; EMR: Electronic medical record; FMF: Family medicine forum; HIPAA: Health Insurance Portability and Accountability Act; PHI: Personal Health Information; REB: Research ethics board

## Competing interests

The authors declare that they have no competing interests.

## Authors' contributions

KEE designed the study, performed data analysis, and contributed to writing the paper. JM designed the study and contributed to writing the paper. KM collected the data, performed data analysis, and contributed to writing the paper. IGG designed the study and contributed to writing the paper. DB designed the study and contributed to writing the paper. EJ coordinated the study and contributed to writing the paper. All of the authors have read and approved the final manuscript.

## Pre-publication history

The pre-publication history for this paper can be accessed here:

http://www.biomedcentral.com/1471-2458/11/454/prepub

## Supplementary Material

Additional file 1 **Summary of Health Reporting Literature**. A literature review of empirical studies evaluating the extent to which providers report communicable diseases where reporting is mandated. Click here for file
