# Supplementary material for: Physician privacy concerns when disclosing patient data for public health purposes during a pandemic influenza outbreak
Source: BMC Public Health. 2011 Jun 9;11:454. doi: 10.1186/1471-2458-11-454 (PMC3130674; doi:10.1186/1471-2458-11-454)
Supplement: Additional file 1 — Summary of Health Reporting Literature. A literature review of empirical studies evaluating the extent to which providers report communicable diseases where reporting is mandated. [file 1471-2458-11-454-S1.PDF]

Appendix A: Summary of Public Health Reporting Literature

In this appendix we provide a summary of the articles that presented evidence on the (un)willingness of physicians to report to public health units even when that reporting was mandatory. An overall summary is provided in Table 1, followed by a discussion of each paper.

This is not a systematic review of all of the evidence, but is intended to demonstrate that reporting even when it is mandated can be quite low.

Author	% of doctors reporting	% of cases reported
Standaert [1]	0.9%	4%
Doyle [2]		9%-99%<br>79% TB, AIDS & STDs<br>49% other diseases
Watkins [3]		100% of inpatient cases, and 79 % of outpatient cases
Thacker [4](reporting on previous studies)		6%-90%
Szecsenyi [5]	Fluctuated from 47.7% to >70%	
Cleere [6]		4.7% gonorrhea cases<br>26.5% syphilis cases*
Rushworth [7]		25% *
Calzavara [8]		85% AIDS cases
Johnson [9]		75.2% AIDS cases
Jenkinson [10]		18.7%
Jones [11]	88 % $^{\alpha}$	55% *
Bernillon [12]		83.6% mandatory system<br>47.6% to the FHDH
Konowitz [13]		28% $^{\alpha}$
Schramm [14]		10% *
Clarkson [15]		40-60% measles 5-25% pertussis
Macleod [16]		83% regional survey<br>76% laboratories
Rothenberg [17]		12%*
Rivest [18]		56.4%*
Scatliff [19]	25% $^{\alpha}$ VD	
Marier [20]		11% hepatitis - 63% tuberculosis<br>35% overall
Gelman [21]		60% syphilis 35% gonorrhea
Allen [22]		54% overall*<br>~40% hepatitis A & pertussis<br>80% measles
Barrett [23]		49%
Bek [24]	50%-54% $^{\alpha}$	
Deeks [25]		46-47%
Harvey [26]	64% $^{\alpha}$	

*****Percent of all reported cases that were reported by physician's offices
$^{\alpha}$ Self reported by physicians

Table 1: Summary of reporting rates when reporting is mandatory.

Article Summaries

In the following sections we provide brief narratives describing each of the articles.

Standaert, Lefkowitz, Horan, et al.,"The reporting of communicable diseases: A controlled study of Neisseria meningitides and Haemophilus influenzae infections" [1]

Standaert and colleagues compared an active laboratory reporting and passive physician reporting surveillance systems for Neisseria meningitides and haemophilius influenzae infections in Tennessee. The passive system sent reporting cards to physicians who were asked to fill them out and return them to the state health department. The laboratory based active system had a coordinator who called the labs bi-weekly to encourage participation and obtain more information on reports. In the passive system, 59 cases of n. meningitides were reported (41 confirmed) and 106 H. influenzae cases (94 confirmed); whereas in the active system 71 cases of N. meningitides were reported and 191 H. influenzae cases. In the passive system, only 0.9% of physicians reported a total of 4% of the cases. The remaining cases were reported by hospitals. Standaert et al. also found significant delays in reporting with the passive system and greater errors in reporting.

Doyle, Glynn, and Groseclose, "Completeness of notifiable infectious disease reporting in the United States: An analytical literature review" [2]

Doyle and colleagues performed a systematic review of the literature on the completeness of notifiable disease reporting in the US. They found 33 studies that quantitatively measured the completeness of reporting by medical providers. They found that reporting completeness ranged from 9-99% in these studies, and was associated with the disease in question. For tuberculosis, AIDS, and sexually transmitted diseases, the reporting rate was higher (79%) than for other diseases (49%). This increase could be due to the serious nature of these diseases, or the greater resources dedicated to treating and preventing these conditions. The studies reviewed also suggest that active surveillance systems tend to have more complete rates of reporting as opposed to passive systems.

Watkins, Lapham, and Hoy, "Use of medical center's computerized health care database for notifiable disease surveillance" [3]

Researchers examined the sensitivity of Lovelace Medical Center's patient tracking and billing system when used for disease surveillance, and the completeness of reporting to authorities. They found that all inpatient laboratory-confirmed cases of the diseases under study, and most out-patient cases were reported to the New Mexico Health and Environmental Department. For outpatient reports, the rate varied by disease with the highest being Shigella (100%) and the lowest being hepatitis A (57%). Overall, 79% of outpatient cases were reported.

Thacker and Berkelman, "Public Health surveillance in the United States" [4]

Authors provide a history of public health surveillance in the US, the purposes of disease surveillance, and tools used for surveillance. They offer several limitations in the practice of public health surveillance, including data gaps created from the underreporting of diseases. Rates of reporting have been estimated to fluctuate from 6% to 90%, depending on the disease being reported. Both measles and AIDS programs have a >90% reporting rate. In one study, it was found that reporting of cases treated by physicians in private offices was greater for shigella, leading to unrepresentative data. Methods to improve the reporting of diseases need further study and evaluation.

Szecsenyi, Uphoff, Ley and Brede, "Influenza surveillance: Experiences from establishing a sentinel surveillance system in Germany" [5]

Reporting on a new German sentinel surveillance network for influenza and its effectiveness in detecting acute respiratory infection (ARI). The sentient network is a network of volunteer doctors that transfers data from physicians' offices weekly, either electronically or by mail for those physicians who did not have a computer system. Reporting rates over the study period fluctuated from 47.7% to >70%.

Cleere, Dougherty, Fiumara, et al. "Physician's Attitudes Toward Venereal Disease Reporting" [6]

This study took place in 1965 and consisted of a physician attitude survey re: reporting of venereal diseases. Researchers found that physicians were selective about the cases that they reported to public health agencies. Doctors self reported that they treated a total of 2063 cases of gonorrhea and 361 cases of syphilis in their offices. Estimated figures based on this sample indicate the total population of doctors would have treated 7202 cases of gonorrhea and 1260 cases of syphilis. However, public health data show that private doctor's offices reported only 340 (4.7%) cases of gonorrhea and 334 (26.5%) of syphilis cases.

In terms of attitudes regarding the reporting of these diseases, it was shown that 56% of the sample was favorable to reporting, 32% were intermediate, and 12% were unfavorable. The most popular reasons for not reporting were:

- to protect the patient from embarrassment and harassment
- confidentiality of the doctor-patient relationship
- patient pressure not to report
- unaware of the reporting requirements
- the disease has been treated/controlled
- another source will report, i.e. laboratory
- reporting is time consuming and onerous

Researchers found that the main problem that worked to reduce rates of reporting was selective reporting by physicians of some cases and not others. There was also implied in physicians' answers, a lack of faith in the public health system to deal with the situation in a proper and discreet manner, and a lack of understanding of the reasons for/importance of reporting.

Rushworth, Bell, Rubin, et al. "Improving surveillance of infectious disease in New South Wales" [7]

Researchers compared a medical practitioner notification system with a laboratory-based system. They found that there was underreporting by physicians as compared to the rates of laboratory reporting. Of 461 total cases reported, 75% were reported by the lab only, while 20.2% were reported by doctors alone, and 4.8% were reported by both sources. Laboratory reports were also found to be more timely and more complete than the practitioners' reports.

Researchers point to reasons found in the literature for underreporting by practitioners. These include patient confidentiality concerns, unaware of the reporting requirements, inadequate feedback by health departments, unclear channels of communication and practical barriers/delays such as issues with delivery of reporting forms.

Calzavara, Coates, Craib, et al. "Underreporting of AIDS cases in Canada: A record linkage study" [8]

Researchers used information from participants in the Toronto Sexual Contact Study (TSCS) and the Vancouver Lymphadenopathy-AIDS study (VLAS) to link to the national registry of reported cases of AIDS. According to the data provided, the proportion of AIDS cases not reported to the registry was 15% - 12% for VLAS and 18% for TSCS.

Johnson, Montano, Wallace, "Using death certificates to estimate the completeness of AIDS case reporting in Ontario in 1985-87" [9]

Researchers used death certificates to measure the completeness of AIDS case reporting in Ontario between 1985 and 1987. They matched the death certificates to cases in the registry using demographic variables. The overall rate of reporting was estimated at 75.2%, with no significant difference across

years. Researchers suggest that the reason for not reporting is the physicians' desire to protect patient confidentiality.

Jenkinson, "Whooping cough: what proportion of cases is notified in an epidemic?" [10]

Researchers requested physicians to estimate the number of patients seen with whooping cough during the period, and compared these estimates to the number of cases reported to the notification system. The number of cases reported in the period was 116, while the estimated number of patients seen was 620. The proportion notified then was only 18.7%.

Jones, Meyer, Garrison, et al. "Physician and Infection Control Practitioner HIV/AIDS reporting characteristics" [11]

The South Carolina Department of Health and Environmental control conducted a survey of physicians to assess reporting of HIV/AIDS in the state. The group surveyed included specialists, primary care physicians, and infection control practitioners (ICPs) most of whom were nurses. 79% of physicians agreed the AIDS should be reportable by name, and 59% indicated that it was the primary care physician's responsibility to report cases. Eighty eight percent of physicians indicated that they report cases of AIDS most of the time or always. Physicians who reported rarely or never were likely to have concerns about confidentiality, legal liability, discrimination, length of the reporting form, and hospital bed size. In this period, 55% of reported cases came from physicians.

Bernillon, Lievre, Pillonel, et al. " Record-linkage between two anonymous databases for a capture-recapture estimation of underreporting of AIDS cases : France 1990-1993" [12]

Researchers examined the completeness of reporting to the French mandatory AIDS surveillance system from 1990-1993 by comparing the reports to the records in the French Hospital Database on HIV infection (FHDH). The total number of cases and the completeness of both systems were estimated using a capture-recapture method. The mandatory system was shown to be much more complete, with an estimated 83.6% of cases reported, whereas the FHDH had a much lower rate of 47.6%. The completeness of the FHDH increase over the period, as more hospitals joined the system.

Konowitz, Petrossian, Rose, "The underreporting of disease and physicians' knowledge of reporting requirements" [13]

Researchers sent a self administered questionnaire to physicians who attended conferences at Mount Sinai medical centers to assess knowledge of reporting requirements. The average score of 15.6 (out of 20) was obtained for physician's knowledge of the diseases in the list of 20 reportable communicable diseases. Only 40 physicians, 24%, knew the correct procedures for reporting, and 47% knew that reporting was required within 24 hours of diagnosis. In terms of reporting, physicians recalled reporting 28% of cases on average.

The reasons for not reporting provided were grouped into these 3 categories:
1. Negative attitude toward reporting - too time consuming, etc.
2. Misconceptions about reporting - i.e. violates doctor-patient confidentiality, patient was treated so no need to report, etc.
3. Lack of knowledge of reporting requirements.

The 2 most common reasons provided were lack of knowledge of how to complete the report, and unawareness that the disease was reportable. Lack of knowledge therefore seems to be the largest obstacle to reporting of diseases.

Schramm, Vogt, Mamolen, "The surveillance of communicable disease in Vermont: Who reports?" [14]

Researchers examined notifiable disease reports received in Vermont to determine the rate of reporting for different sources. They found that 71% of reports were received from laboratories, whereas only 10% were received from physician's offices.

They also surveyed physicians to find out more about their reporting habits, and found that only 18% of physicians indicated that they always reported cases of notifiable diseases.

Clarkson and Fine, "The efficiency of measles and pertussis notification in England and Wales" [15]

Researchers looked at reports for measles and pertussis in England and Wales, reported to the Office of Population Censes and Surveys. They used the Hospital Inpatient Enquiry (HIPE) to obtain estimates of the annual number of hospitalized cases and obtained birth and population estimates from the Registrar General and OPCS.

Prior to vaccination for these diseases, they estimated that the total number of cases should approximate the annual number of births. After the vaccination program began, it was assumed that all children who were not vaccinated contracted the disease. The results were that 40-60% of measles cases were estimated to have been reported, and 5-25% of pertussis infections in the period under study.

Macleod, "Haemophilus influenzae: the efficiency of reporting invasive disease in England and Wales" [16]

Researchers compared a special regional survey with customary laboratory reporting in measuring cases of Haemophilius influenzae prior to the introduction of routine vaccination. The survey results outnumbered the laboratory reporting, with 541 cases reported vs. 383 cases from labs. Comparing the reports, researchers found that 111 cases were reported to the CDSC by laboratories that were not captured by the survey, so the total number of reports was then 652 cases. Underreporting was estimated at 17% for the regional survey, and 24% from the laboratories. Underreporting varied by region for both sources, with a maximum variance of 25% for the survey and 26% between reporting laboratories in different regions.

Rothenberg, Bross, Vernon, "Reporting of gonorrhea by private physicians: a behavioral study" [17]

Researchers studies reporting patterns of physicians to state health departments in the US for the sexually transmitted disease, gonorrhea. They assert that in the Denver Metropolitan area, physicians report 12% of the total number of cases reported.

A sample of 1594 physicians was randomized 5 study groups and a control group. The 5 study groups all received a different intervention to test the hypothesized reasons for underreporting: 1. Lack of saliency, 2. Patient interference, 3. Increase in privileged information (i.e. need to follow up with patient's contacts), 4. Insufficient incentives, 5. Administrative obstacles. The corresponding interventions were: 1. A letter requesting help in the control effort through continued reporting of all venereal diseases, 2. A letter expressing understanding re: the conflict of interest between protecting patient privacy and the public health, 3. A reminder letter sent with a return postcard asking for the reasons for not reporting and the number of cases seen, 4. A reward was sent to reporting physicians of a thank you note and a reprint on sexually transmitted diseases, and lastly 5. Physicians' offices contact by telephone and arrangements were made to periodically contact a nurse or support staff in regards to disease reporting. The only group to show a significant increase in reporting was the telephone group in which reporting more than doubled. This is consistent with other reports that an active surveillance system can improve reporting of communicable diseases

Rivest, Sagot, Bedard, "Evaluation of the completeness of reporting of invasive meningococcal disease" [18]

Researchers examined the reporting of meningococcal disease in the province of Quebec, by comparing the reportable diseases central registry (MADO) and the hospital discharge data registry (MED-ECHO) for patients hospitalized in Montreal. They found 58 cases reported to the MADO, of which 51 were confirmed by laboratory findings, and 64 cases were found in the hospital discharge registry but 13 were excluded due to errors. Of the 51 hospital reports, 47 were confirmed by laboratory testing. The total of cases of meningococcal disease in the area was estimated at 58 cases, so the overall completeness of reporting was 94.8%. Physicians were the first to report 56.4% of the cases reported.

Scatliff, "Survey of venereal disease treated by Manitoba physicians in 1972" [19]

Scatliff performed a survey of Manitoba physicians to learn more about the treatment and reporting of venereal disease. The research team surveyed 829 doctors and of these, 548 reported that they had seen cases of venereal disease in their practices during the period. Of those, 140 (25%) self-reported that they notify public health of all cases presented.

In terms of reasoning, doctors reported that confidentiality, or fear that confidentiality would be broken by the public health authority, was a great deterrent for reporting. Other reasons include design of the reporting form, and lack of intraprofessional communication and feedback from the public health authority.

Marier, "The reporting of communicable diseases" [20]

Marier evaluates the effectiveness of the standard passive reporting methods used in Washington for the reportable diseases viral hepatitis, H. influenzae and meningococcal meningitis, salmonellosis, shigellosis and tuberculosis. He found an overall reporting rate of 35%, stratified by disease. Hepatitis has the lowest percentage of cases reported (11%), while tuberculosis had the highest rate (63%).

Marier also reports on a survey of physicians' attitudes conducted by the National Opinion Research Centre in 1966, which provided various reasons for underreporting of notifiable diseases. The greatest reasons for non-reporting were a lack of awareness of reporting requirements, concerns about the confidentiality of venereal disease reports, and a feeling that reporting of some diseases of lesser concern (i.e. measles) was irrelevant.

A copy of the original survey by the National Opinion Research Centre could not be obtained.

Gelman, "Current status of venereal disease in New York City: A survey of 6,649 physicians in solo practice" [21]

Researchers surveyed 6,649 physicians in New York City in various types of private practice that would treat venereal diseases. They received 5,046 responses, with 1960 reporting that they had treated venereal disease in the period. These physicians estimated that they treated 2869 cases of syphilis and 15,573 cases of gonorrhea. When compared to the numbers reported to the Health Department, and adjusting for non-participating physicians, no more than 60% of syphilis cases and 35% of gonorrhea cases were estimated to have been reported to the health department.

The physicians surveyed suggested some methods to improve disease control which help give light to some reasons for underreporting of venereal disease. These include a hesitation to provide patients' names, and concerns in regard to how the Health Department dealt with reports/follow-ups.

Allen & Ferson, "Notification of infectious diseases by general practitioners: a quantitative and qualitative study" [22]

Authors performed an audit of the records held by the Public Health Unit in South Eastern Sydney to examine from where the notifications originated. They found that 54% of the cases examined were notified by general practitioners. In the three difference diseases studies, laboratories far out-reported GPs, except for in the case of measles where GPs reported 81% vs. 25% of cases reported by the laboratories.

In the qualitative portion of the study, the researchers found that GPs were relying on the laboratory to send notification, which also provided a loop-hole in terms of any privacy concerns they may have regarding reporting. Doctors also reported dissatisfaction with the reporting process at the time, a lack of remuneration for the time spent, and hesitation to notify suspected cases due to consequences for the patient.

Barrett & Lau, "Incompleteness of statutory notification of bacterial gastro-intestinal infection" [23]

Researchers identified patients with bacterial gastro-intestinal infection from fecal samples submitted by doctors. Infected patients were looked up in the notification records of the Health Authority and doctors were sent a questionnaire if no notification had been received after a period of 6 weeks. Overall, 49% of cases were reported: 51% of all cases seen by a GP, and 47% of cases seen at hospital. The most common reasons for not notifying provided in the survey included: "forgot to notify", "the result of the specimen was not received", and "somebody else was thought to have made the notification"[23].

Bek, Loney & Levy, "Notification of infectious diseases by general practitioners in New South Wales" [24]

Authors examined the knowledge and opinions of GPs in regards to public health reporting in relation to the introduction of the Public Health Act in New South Wales. The authors surveyed GPs and found that 50% of GPs reported notifying "almost always, or always" before the Act was introduced and 54% reported the same after the Act was introduced. Reported reasons for not notifying cases of infectious disease decreased in frequency after the Act was introduced, particularly for those who were unaware of which diseases to notify (38% before vs. 23% after) and those who said that they did not have the necessary forms (22% before vs. 11% after).

Deeks, Serres, Boulianne, et. al., "Failure of physicians to consider the diagnosis of pertussis in children" [25]

Researchers studied notification of pertussis diagnosis in children. Using two clinical case definitions, the surveillance case definition (SCD) and the World Health Organization case definition (WHO), the authors included in the study children who met at least one of the case definitions and who had seen a doctor for their cough. They found that only 46-47% of diagnoses were reported to the surveillance system.

Harvey, Park, Palmer and Peters, "Infectious disease notification – a neglected legal requirement" [26]

In a questionnaire sent to hospital doctors, 64% of respondents indicated that they had notified cases of infectious disease. Eighty-two percent of the respondents knew that notification was compulsory, but on 60% knew who should be notified and 70% said that they did not know how to obtain a reporting form. The doctors also reported being unaware that certain diseases were notifiable: 55% did not know about meningitis, 53% did not know about measles and 39% did not know about whooping cough or food poisoning.

References

1. Standaert S, Lefkowitz L, Horan J, Hutcheson R, Schaffner W. The reporting of communicable diseases: A controlled study of Neisseria meningitides and Haemophilus influenzae infections. Clinical Infectious Diseases, 1995; 20:30-36.
2. Doyle T, Glynn K, Groseclose S. Completeness of notifiable infectious disease reporting in the United States: An analytical literature review. American Journal of Epidemiology, 2002; 155(9):866-874.
3. Watkins M, Lapham S, Hoy W. Use of medical center's computerized health care database for notifiable disease surveillance. American journal of Public Health, 1991; 81(5):637-639.
4. Thacker S, Berkelman R. Public Health surveillance in the United States. Epidemiologic Reviews, 1988; 10:164-190.
5. Szecsenyi J, Uphoff H, Ley S, Brede D. Influenza surveillance: Experiences from establishing a sentinel surveillance system in Germany. Journal of Epidemiological Community Health, 1995; 49 (suppl 1):9-13.
6. Cleere R, Dougherty W, Fiumara N, Jenike C, Lentz J, Rose N. Physician's Attitudes Toward Venereal Disease Reporting. JAMA, 1967; 202(10):117-122.
7. Rushworth R, Bell S, Rubin G, Hunter R, Ferson M. Improving surveillance of infectious disease in New South Wales. The Medical Journal of Australia, 1991; 154:828-831.
8. Calzavara L, Coates R, Craib K, Math M, Schechter M, Le T, Nault P, Elmslie K. Underreporting of AIDS cases in Canada: A record linkage study. CMAJ, 1989; 142(1):36-39.
9. Johnson R, Montano B, Wallace E. Using death certificates to estimate the completeness of AIDS case reporting in Ontario in 1985-87. CMAJ, 1989; 141:537-540.
10. Jenkinson D. Whooping cough: what proportion of cases is notified in an epidemic? BMJ, 1983; 287:185-186.
11. Jones J, Meyer P, Garrison C, Kettinger L, Hermann P. Physician and Infection Control Practitioner HIV/AIDS reporting characteristics. American journal of Public Health, 1992; 82(6):889-891.
12. Bernillon P, Lievre L, Pillonel J, Laporte A, Costagliola D, Clincal Epidemiology Group C. Record-linkage between two anonymous databases for a capture-recapture estimation of underreporting of AIDS cases : France 1990-1993. International Journal of Epidemiology, 2000; 29:168-174.
13. Konowitz P, Petrossian G, Rose D. The underreporting of disease and physicians' knowledge of reporting requirements. Public Health Reports, 1984; 99(1):31-35.
14. Schramm M, Vogt R, Mamolen M. The surveillance of communicable disease in Vermont: Who reports? Public Health Reports, 1991; 106(1):95-97.
15. Clarkson J, Fine P. The efficiency of measles and pertussis notification in England and Wales. International Journal of Epidemiology, 1985; 14(1):153-168.
16. Macleod C. Haemophilus influenzae: the efficiency of reporting invasive disease in England and Wales. CDR Review, 1994; 4(2):13-16.
17. Rothenberg R, Bross D, Vernon T. Reporting of gonorrhea by private physicians: a behavioral study. AJPH, 1980; 70(9):983-986.
18. Rivest P, Sagot B, Bedard L. Evaluation of the completeness of reporting of invasive meningococcal disease. Revue Canadienne de Sante Pubique, 1999; 90(4):250-252.

19. Scatliff J. Survey of venereal disease treated by Manitoba physicians in 1972. CMAJ, 1974; 110:179-182.
20. Marier R. The reporting of communicable diseases. American Journal of Epidemiology, 1977; 105(6):587-590.
21. Gelman A, Vandow J, Sobel N. Current status of venereal disease in New York City: A survey of 6,649 physicians in solo practice. American journal of Public Health, 1963; 53:1903-1918.
22. Allen C, Ferson M. Notification of infectious diseases by general practitioners: a quantitative and qualitative study. Med J Aust., 2000; 172(7):325-8.
23. Barrett P, Lau Y. Incompleteness of statutory notification of bacterial gastro-intestinal infection. Public Health, 1997; 111(3):183-5.
24. Bek M, Lonie C, Levy M. Notification of infectious diseases by general practitioners in New South Wales. Survey before and after the introduction of the Public Health Act 1991 (NSW). Med J Aust., 1994; 161(9):538-41.
25. Deeks S, De Serres G, Boulianne N, Duval B, Rochette L, Dery P, Halperin S. Failure of physicians to consider the diagnosis of pertussis in children. Clin Infect Dis., 1999; 28(4):840-6.
26. Harvey I, Palmer S, Peters T. Infectious disease notification - a neglected legal requirement. Health Trends, 1991; 23:73-74.
